# Trends of Cardiac Complaints in Pediatric and Young Adult Populations During the COVID-19 Pandemic

**DOI:** 10.3390/jcdd12040138

**Published:** 2025-04-07

**Authors:** Emily M. Ferraro, Madeline I. Dorr, Cade M. Nylund, Apryl Susi, Elizabeth Hisle-Gorman, Michael Rajnik, Brian N. Hughes

**Affiliations:** 1 Department of Pediatrics, Walter Reed National Military Medical Center, Bethesda, MD 20814, USA; eferraro817@gmail.com; 2 Department of Pediatrics, Uniformed Services University, Bethesda, MD 20814, USA; maddyisabelledorr@gmail.com (M.I.D.); apryl.susi.ctr@usuhs.edu (A.S.); elizabeth.hisle-gorman@usuhs.edu (E.H.-G.); 3 Henry M. Jackson Foundation for the Advancement of Military Medicine, Bethesda, MD 20817, USA

**Keywords:** SARS-CoV-2, palpitations, syncope, chest pain, pediatrics

## Abstract

The COVID-19 pandemic had a significant impact on the physical and mental health of all age groups. While many studies have evaluated the serious cardiac manifestation associated with multisystem inflammatory syndrome of children, there are less studies evaluating how the COVID-19 pandemic impacted the presentation of less severe symptomatic cardiac manifestations. This large retrospective cross-sectional study examined the impact of the COVID-19 pandemic on the cardiovascular health of patients 1 to 24 years of age by assessing care presentation trends for chest pain, palpitations, and syncope for patients in the military health system. Overall, chest pain had the highest rate of presentation when compared to syncope and palpitations. There was a significant decrease in the rate of incidence for all three cardiac symptoms during the first year of the COVID-19 pandemic compared to the pre-COVID-19 period. When comparing the second year of the COVID-19 pandemic to the pre-COVID-19 period, there was a significant decrease in chest pain and syncope but a slight increase in palpitations. Overall, our results showed that these common cardiac presentations did not greatly increase during the COVID-19 pandemic.

## 1. Introduction

The emergence of the SARS-CoV-2 virus in March 2020 created an unparalleled global pandemic. Widespread shelter-in-place orders and lockdowns led to the closure of schools, cessation of extracurricular activities, and decreased social interactions. The immediate and lasting impact on cardiovascular health is a topic of ongoing research and relevant to both adult and pediatric medical providers.

Multisystem Inflammatory Syndrome of Children (MIS-C), a condition resulting from post-infection inflammation and vaccine-related myocarditis and pericarditis, are cardiac conditions occurring in pediatric populations during the COVID-19 pandemic and have been heavily researched [1,2,3,4]. Fewer studies have examined the impact of the COVID-19 pandemic on overall pediatric cardiovascular health and less severe symptomatic cardiac presentations [3,4,5]. We identified a gap in research looking into pediatric cardiac symptoms that were not related to MIS-C, myocarditis, or pericarditis that occurred during the COVID-19 pandemic. The direct and indirect biopsychosocial effects of living through the SARS-CoV-2 pandemic remain topics of interest. Indirect biopsychosocial factors of the pandemic may include decreased healthy eating behaviors, decreased physical activity, increased obesity rates, and increased isolation, leading to higher prevalence of anxiety and depression [6,7,8,9]. A 2020 study of 782 pediatric patients presenting to the outpatient clinic found that only 1% of patients were determined to have chest pain from cardiac etiology, while musculoskeletal (33%) and psychogenic (28.4%) disorders were significantly more common causes [10].

Determining the effects of the SARS-CoV-2 virus and pandemic-associated factors on cardiovascular health is challenging. Evaluating changes in symptomatic cardiac presentations between pre- and active pandemic periods is one method that has been used in the recent literature to assess clinical trends in cardiovascular health. A single-center study in 2022 evaluated symptomatic presentations of chest pain, chest tightness, and palpitations in children and adolescents in the pre- vs. active pandemic time periods and assessed if they were thought to be organic (cardiac) or non-organic (anxiety or depression) [11]. The study found increased presentation between January and April 2021 compared to the pre- and early COVID-19 periods (January–April 2020). Furthermore, a larger percentage of symptoms were of non-organic etiology during the post-COVID-19 pandemic period compared to the pre-pandemic period [11]. To our knowledge, no other large-scale studies exist in the pediatric literature that highlights the need for additional large-scale, national studies.

To address this gap in current understanding, we sought to examine incidence rates of three common pediatric cardiac complaints that often require cardiology consultation (chest pain, palpitations, and syncope) before and during the COVID-19 pandemic in a large, retrospective national military-wide study. We wanted to focus on individuals with cardiac symptoms that could not be attributed to myocarditis, pericarditis, endocarditis, or cardiomyopathy. We hypothesized that rates of presentation of cardiac symptoms during the pandemic would increase due to factors such as the direct impact of infection, cardiac deconditioning, and symptomatic anxiety.

## 2. Materials and Methods

This retrospective monthly repeated cross-sectional study used data from the Military Health System (MHS) Data Repository. The MHS Data Repository (MDR) includes all outpatient care records, inpatient care records, procedure records, demographic information, and enrollment and eligibility data for the estimated 9.2 million military beneficiaries, including care at both military and civilian facilities. TRICARE is the health insurance program for the United States of America’s active duty military service members, active duty family members, military retirees, and retiree family members. TRICARE insurance covers care delivered in Military Treatment Facilities as well as purchased care from civilian health institutions where a large amount of emergency and urgent medical care is delivered [12]. Data was extracted from the MDR for inpatient and outpatient records of service members and military dependents 1 to 24 years of age. The International Classification of Disease (ICD) 10th revision codes were used to identify incident cardiology symptoms diagnoses.

TRICARE beneficiaries enrolled for a given month from January 2019 to May 2022 were eligible for inclusion. An individual was excluded if they were pregnant at any time during the study period. Individuals were also excluded if they had a diagnosis of myocarditis, pericarditis, endocarditis, or cardiomyopathy anytime during the study period. Monthly counts of incident chest pain, palpitations, and syncope were calculated. Incidence was defined as the first diagnosis among those without a previous diagnosis within the preceding 12 months, which started in January 2018. If an individual had an incident diagnosis, they were then excluded from subsequent months. Individuals less than 1 year old or older than 25 years in a given month were excluded from that month’s count.

An individual’s age, sex, military rank (or military sponsor rank), and geographic region of care were extracted from the individual’s enrollment record. Age was grouped into 1 to 10 years, 11 to 17 years, and 18 to 24 years. Rank was categorized as junior enlisted (pay grades E1–E4), senior enlisted (pay grades E5–E10), or officer rank. Ranks were used to serve as a surrogate for education and income. The geographic region of care is based on the TRICARE definitions of North, South, West, and outside the continental USA (OCONUS), which includes Alaska, Hawaii, and overseas locations (OCONUS). The time period was defined as pre-COVID-19 (January 2019–February 2020), COVID-19 Year 1 (June 2020–May 2021), and COVID-19 Year 2 (June 2021–May 2022). The months of March 2020–May 2020 were excluded due to widespread clinic closures.

The median percent of the monthly patient population was calculated in each demographic category. Rates per 100,000 individuals a month were determined for each cardiac complaint. Incidence rate ratios by COVID-19 time period were calculated using Poisson regression analysis. Three separate Poisson regression models included chest pain, palpitations, and syncope as dependent variables and COVID-19 periods (reference = pre-COVID-19), age group (reference = 1 to 10 years), sex (reference = male), rank (reference = junior enlisted), and region (reference = North) as independent variables. SAS version 9.4 (Cary, NC, USA) was used for statistical analysis. *p*-values of <0.05 were considered significant. The study protocol was reviewed and deemed no more than minimal risk to human subjects by the institutional review board of the Uniformed Services University of the Health Sciences, Bethesda, MD, USA.

## 3. Results

Our study included an average of 2.7 million individuals per month. At the time of first inclusion, a total of 4.4 million unique individuals were in our study. The study population was divided into four primary demographic categories: age, sex, sponsor military rank, and region (Table 1). The late adolescent age group was included in the study population to capture young active duty service members. There was a male-to-female predominance (57% to 43%). Enlisted (senior and junior) service members and their dependents (i.e., spouses or children) made up nearly 80% of the study population with the remainder having an officer rank, which is consistent with known active duty rank distribution [13]. West, North, and South TRICARE regions were equally distributed, with a small percentage in the OCONUS region.

**Figure 1:**
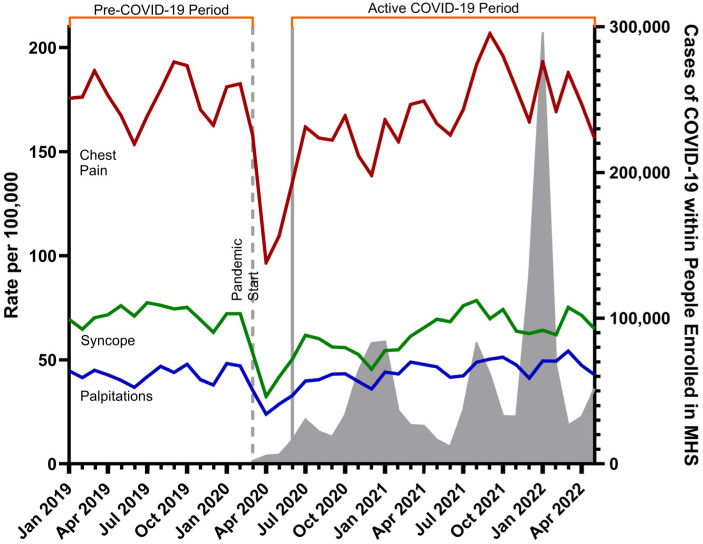
Monthly incidence rates of cardiac symptoms across study period.

When comparing COVID-19 Year 1 to the pre-COVID-19 period, there was a significant decrease in the rate of incidence for chest pain, palpitations, and syncope (Figure 1). During the COVID-19-Year 1 period, rates of all three symptoms dropped as COVID-19 infections peaked in December 2020. When comparing COVID-19 Year 2 to the pre-COVID-19 period, there was a significant decrease in chest pain and syncope, but a slight increase in palpitations. Chest pain had the highest rates of presentation when compared to syncope and palpitations. There was a spike in incidence of chest pain between November 2021–February 2022 which aligned with an acute increase in cases of COVID-19.

Rate per 100,000 people for each of the three cardiac complaints across the entire study period with background rates of COVID-19 infection within the Military Health System (MHS) are overlayed in gray. Rates of palpitations are denoted with the blue line, rates of syncope with the green line, and rates of chest pain with the red line. The vertical dashed line indicates the start of the COVID-19 pandemic. COVID-19 Year 1 starts in June 2020 through June 2021 and is delineated between the first two solid gray lines. COVID-19 Year 2 starts in July 2021 through May 2022 and begins at the second solid gray line. The months of March 2020–May 2020 were excluded due to widespread clinic closures.

Table 2 displays the cardiac care rate ratios of presentation for the four demographic categories. Incident rate ratios of all three symptoms were higher in both the 18–24 year age group and the 11–17 year age group compared to the 1–10 year age group. Females had statistically significant higher rates of incidence for chest pain, palpitations, and syncope when compared to males. With respect to military rank, officers, and those with an officer sponsor showed decreased incident rates of chest pain and syncope, but increased incident rates of palpitations when compared to junior enlisted populations. Senior enlisted populations had higher rates of chest pain and palpitations.

## 4. Discussion

We assessed a large pediatric and young adult population with widespread geographic distribution and found decreased rates of presentation for chest pain and syncope during the COVID-19 pandemic compared to pre-pandemic rates. Rates of presentation for palpitations decreased during the first COVID-19 year and increased during the second year. Our results address a scarcity of data surrounding the impact of the COVID-19 pandemic on commonly presented pediatric cardiac complaints and indicate that initial decreases in the presentation of these symptoms were multifactorial. As care became more accessible, the result was a return to baseline levels of syncope and palpitations, with a slight increase in the presentation for chest pain that was associated temporally with an increase in SARS-CoV-2 infection in the MHS. Overall, our results showed that common cardiac presentations were not greatly increased during this era.

We postulate multiple potential influencing factors contributing to our findings. First, widespread school closures and cessation of extracurricular sports and activities led to decreased participation in physical activity that often precipitates the symptoms we assessed. A 2020 study including children from 35 states and the District of Columbia found that parents perceived a decrease in children’s physical activity and an increase in sedentary behavior compared to the pre-COVID-19 period and the early part of the pandemic, with a more significant impact on children age 9–13 years old compared to children age 5–8 years old [14]. Second, hesitancy and barriers to seeking care during the pandemic period may have precluded presentation of more mild symptoms when compared to the pre-pandemic period. Similarly, the impact of both parental and patient anxiety during the pandemic may have impacted willingness to seek care for less severe symptoms. A study during the COVID-19 pandemic found that 36% of caregivers believed that they had barriers to obtaining care, including fear of contracting COVID-19 and government instruction to avoid health care visits for minor problems [15]. A military study found a statistically significant decrease in median monthly well visit rates when comparing the pre-COVID-19 and COVID-19 periods [16].

Results for the various demographic groups were heterogeneous but may highlight interesting trends warranting further evaluation. When compared to males, females across the entire study population showed higher rates of presentation for all three symptoms when compared to males. This trend is consistent with the current literature and those studies also showed that females have better outcomes compared to males [17,18,19]. Increased presentations among the older age groups (18–24 and 11–17) compared to the 1–10 age group reflected known age-related trends of presentation for palpitations [20]. Our examination of a large military population also showed interesting trends between service members and dependents of enlisted rank that likely require further investigation regarding access to care across military ranks.

When interpreting our results with the rates of COVID-19 infection, there was a known increase in disease in children during the Delta and Omicron variant-dominant periods, which may align with increased incidence of chest pain during those time periods (November 2021–February 2022) [21]. These data are the only findings we have to suggest that the increase in cardiac symptoms could be associated with SARS-CoV-2 infection.

Previous pediatric studies focused on diagnostic findings, level of vasopressor support, and cardiac manifestations (myocarditis, coronary artery dilation, or heart failure) associated with COVID-19 infection or MIS-C [3,4,5]. In these studies, the presenting cardiac symptoms were not specific. Our study is the first large-scale COVID-19 study to focus on individuals with symptoms of chest pain, syncope, or palpitations that could not be attributed to myocarditis, pericarditis, endocarditis, or cardiomyopathy.

We identified several limitations to our study. First, individual COVID-19 infection status and COVID-19 vaccination status were not examined in our study population. Further analysis of symptomatic cardiac presentations in vaccinated patients would likely yield important clinical trends. Second, our subgroup analyses for the four demographic categories (age, sex, military rank, and geographic region) were not assessed in the pre- vs. post-pandemic periods but rather across the entire data collection period, limiting our ability to assess differences in these populations in the pre- vs. active COVID-19 periods. Third, COVID-19 variants do not present themselves homogeneously and we were unable to assess their role in the findings in our study.

## 5. Conclusions

Our study provides the groundwork for characterizing the impacts of the global COVID-19 pandemic on pediatric cardiovascular health. While there was immense concern for the cardiotropic nature of SARS-CoV-2 and the subsequent immunizations, our findings suggest that common cardiac presentations were not greatly increased during this era. Further research is required to better define pandemic-associated clinical trends in cardiac health across ages, genders, and socioeconomic factors. This knowledge is critical to promoting health and wellness in the pediatric and young adult populations.

## Figures and Tables

**Table 1:** Study demographics by Median Monthly Percent.

		Median Monthly Percent
**Age**	1–10 years old	35%
	11–17 years old	38%
	18–24 years old	27%
**Sex**	Female	43%
	Male	57%
**Self or Sponsor Rank**	Junior Enlisted	26%
	Officer	21%
	Senior Enlisted	53%
**Region**	North	31%
	Other	7%
	South	32%
	West	30%

**Table 2:** Cardiology outcome-adjusted rate ratios.

		Chest Pain		Palpitations		Syncope	
		RR	95% CI	RR	95% CI	RR	95% CI
Time Period	COVID-19 Year 1 vs. pre-COVID-19	0.87	(0.86–0.88)	0.94	(0.92–0.96)	0.76	(0.75–0.78)
	COVID-19 Year 2 vs. pre-COVID-19	0.97	(0.96–0.98)	1.03	(1.01–1.05)	0.91	(0.90–0.93)
Age (in years)	11 to 17 vs. 1 to 10	2.52	(2.47–2.56)	3.46	(3.34–3.58)	4.35	(4.23–4.47)
	18 to 24 vs. 1 to 10	4.59	(4.52–4.66)	7.91	(7.65–8.18)	5.18	(5.04–5.31)
Sex	Female vs. Male	1.24	(1.23–1.25)	1.78	(1.75–1.81)	1.89	(1.86–1.92)
Sponsor Rank	Officer vs. Jr. Enlisted	0.82	(0.80–0.83)	1.26	(1.23–1.30)	0.85	(0.83–0.91)
	Sr. Enlisted vs. Jr. Enlisted	1.03	(1.02–1.04)	1.32	(1.29–1.36)	0.89	(0.87–0.91)
Region	Other vs. North	0.85	(0.83–0.86)	0.70	(0.67–0.74)	0.74	(0.71–0.76)
	South vs. North	1.14	(1.13–1.16)	1.09	(1.07–1.12)	1.08	(1.06–1.10)
	West vs. North	1.00	(0.99–1.02)	0.90	(0.88–0.93)	0.97	(0.95–0.99)

RR = Rate Ratios; CI = Confidence Interval.

## Data Availability

Restrictions apply to the availability of these data. Data obtained are available from the Defense Technical Information Center (https://discover.dtic.mil/) with the appropriate approval.
